# {4-Bromo-2-[(2-{(ethyl­sulfan­yl)[(2-oxido­benzyl­idene-κ*O*)amino-κ*N*]methyl­idene}hydrazinyl­idene-κ*N* ^1^)meth­yl]phenolato-κ*O*}(ethanol-κ*O*)dioxido­uranium(VI)

**DOI:** 10.1107/S1600536813014669

**Published:** 2013-06-08

**Authors:** Roberto Centore, Mehdi Ahmadi, Andrea Peluso

**Affiliations:** a Dipartimento di Scienze Chimiche, Università degli Studi di Napoli ’Federico II’, Complesso di Monte S. Angelo, Via Cinthia, 80126 Napoli, Italy; b Department of Chemistry, Payame Noor University, 19395–4697, Tehran, Iran; c Dipartimento di Chimica e Biologia, Università di Salerno, Via Ponte don Melillo, 84084 Salerno, Italy

## Abstract

In the title complex, [U(C_17_H_14_BrN_3_O_2_S)O_2_(C_2_H_5_OH)], the U^VI^ cation has a distorted penta­gonal–bipyramidal environment with the penta­gonal plane defined by two N and two O atoms of the tetra­dentate Schiff base ligand and the O atom of the ethanol mol­ecule. Two oxide O atoms occupy the axial positions. The azomethine C=N group and the Br atom are disordered over two positions in a 0.8356 (18):0.1644 (18) ratio. The ethyl­thiolyl group is disordered over three conformations in a 0.8356 (18):0.085 (6):0.079 (6) ratio, and the ethanol ligand is also disordered over three orientations in a 0.470 (16):0.277 (19):0.253 (18) ratio. In the crystal, mol­ecules form centrosymmetric dimers through hydrogen bonding between ethanol O—H donors and phenolate O-atom acceptors. Weak C—H⋯O inter­actions consolidate the crystal packing.

## Related literature

For semiconductor materials containing heterocycles, see: Centore, Ricciotti *et al.* (2012 ▶). For the structural and theoret­ical analysis of conjugation in sulfur-containing metal­organic compounds, see: Takjoo *et al.* (2011 ▶); Takjoo & Centore (2013 ▶). For recent examples of hydrogen bonding in crystals, see: Centore *et al.* (2013 ▶). For the structure of a related complex, see: Takjoo *et al.* (2012 ▶).
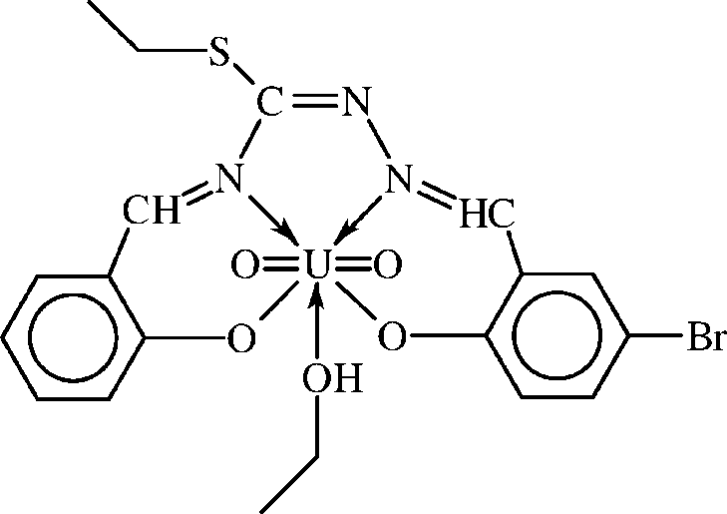


## Experimental

### 

#### Crystal data

[U(C_17_H_14_BrN_3_O_2_S)O_2_(C_2_H_6_O)]
*M* *_r_* = 720.38
Triclinic, 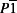

*a* = 10.3720 (17) Å
*b* = 11.1380 (14) Å
*c* = 11.167 (1) Å
α = 69.428 (10)°
β = 86.870 (11)°
γ = 70.379 (10)°
*V* = 1134.7 (3) Å^3^
*Z* = 2
Mo *K*α radiation
μ = 9.04 mm^−1^
*T* = 293 K
0.40 × 0.20 × 0.20 mm

#### Data collection

Bruker–Nonius KappaCCD diffractometer
Absorption correction: multi-scan (*SADABS*; Bruker, 2001 ▶) *T*_min_ = 0.123, *T*_max_ = 0.265
15923 measured reflections
5207 independent reflections
4347 reflections with *I* > 2σ(*I*)
*R*_int_ = 0.045

#### Refinement

*R*[*F* ^2^ > 2σ(*F* ^2^)] = 0.029
*wR*(*F* ^2^) = 0.068
*S* = 1.08
5207 reflections
306 parameters
53 restraints
H-atom parameters constrained
Δρ_max_ = 1.02 e Å^−3^
Δρ_min_ = −1.22 e Å^−3^

### 

Data collection: *COLLECT* (Nonius, 1999 ▶); cell refinement: *DIRAX/LSQ* (Duisenberg *et al.*, 2000 ▶); data reduction: *EVALCCD* (Duisenberg *et al.*, 2003 ▶); program(s) used to solve structure: *SIR97* (Altomare *et al.*, 1999 ▶); program(s) used to refine structure: *SHELXL97* (Sheldrick, 2008 ▶); molecular graphics: *ORTEP-3 for Windows* (Farrugia, 2012 ▶) and *Mercury* (Macrae *et al.*, 2006 ▶); software used to prepare material for publication: *WinGX* (Farrugia, 2012 ▶).

## Supplementary Material

Crystal structure: contains datablock(s) global, I. DOI: 10.1107/S1600536813014669/cv5416sup1.cif

Structure factors: contains datablock(s) I. DOI: 10.1107/S1600536813014669/cv5416Isup2.hkl

Additional supplementary materials:  crystallographic information; 3D view; checkCIF report

## Figures and Tables

**Table 1:** Hydrogen-bond geometry (Å, °)

*D*—H⋯*A*	*D*—H	H⋯*A*	*D*⋯*A*	*D*—H⋯*A*
O5*A*—H5*A*⋯O1^i^	0.78	1.89	2.618 (5)	155
C7—H7⋯O3^ii^	0.93	2.53	3.235 (6)	133
C6—H6⋯O3^ii^	0.93	2.63	3.368 (6)	137
C11—H11⋯O4^iii^	0.93	2.66	3.443 (7)	143
C19*A*—H19*B*⋯O4^i^	0.96	2.58	3.451 (19)	151

Symmetry codes: (i) 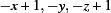; (ii) 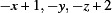; (iii) 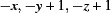.

**Fig. 1.**
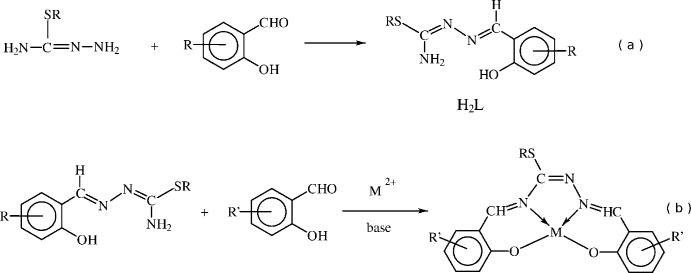
(a) General synthesis of isothiosemicarbazones; (b) Template reaction between an isothiosemicarbazone, a substituted salicylaldehyde and a metal ion affording a complex with a tetradentate isothiosemicarbazate ligand.

**Fig. 2.**
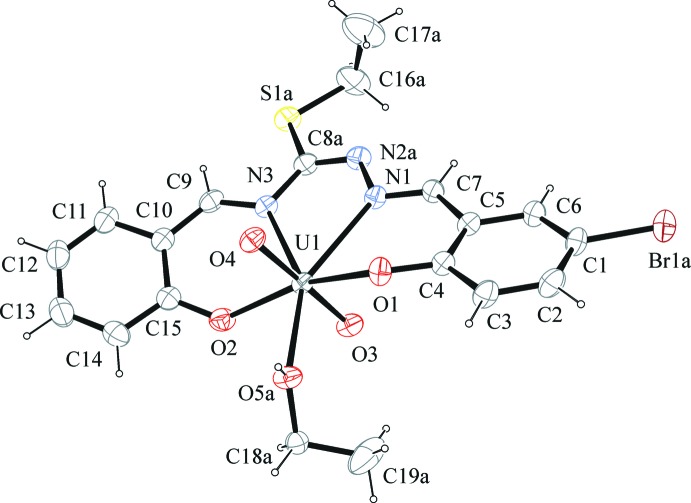
View of (I) showing the atomic numbering and 30% probability displacement ellipsoids. For the disordered atoms, only the major components are shown.

**Fig. 3.**
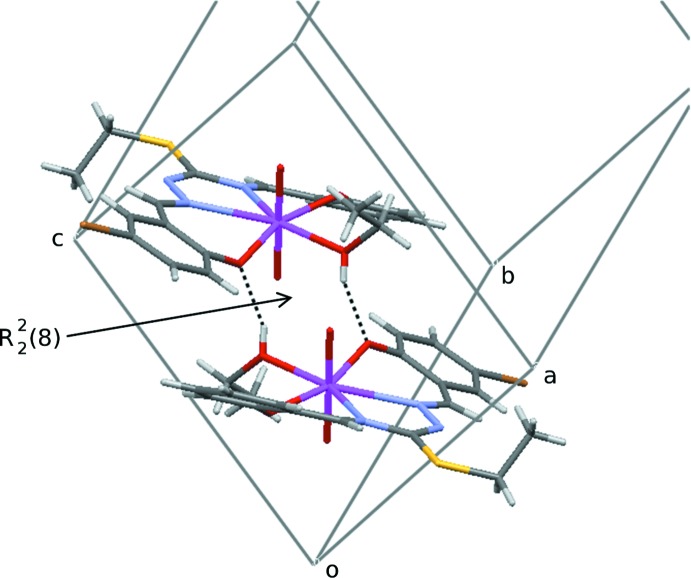
Centrosymmetric H bonded dimer in (I). H bonds are represented by dashed lines. For the disordered atoms, only the major components are shown.

**Fig. 4.**
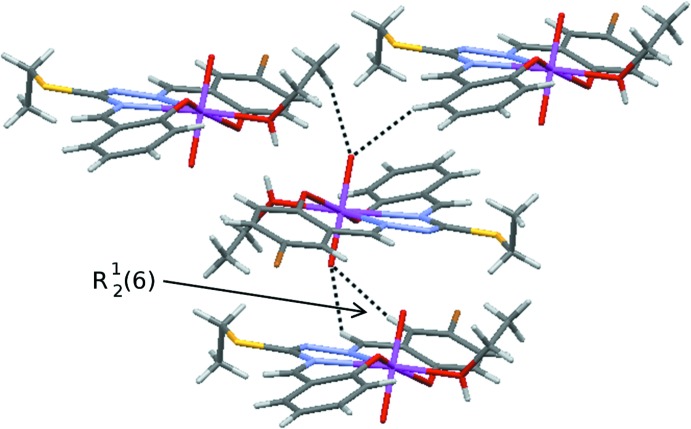
Weak C—H···O interactions (dashed lines), involving oxygen atoms of the uranyl group. For the disordered atoms, only the major components are shown.

## supplementary crystallographic information

### Comment

Condensation between *S*-alkyl-isothiosemicarbazides and salicylaldehyde analogues affords Schiff bases, called isothiosemicarbazones, that can act as tridentate donor ligands, Fig. 1 (*a*). Isothiosemicarbazones are versatile ligands because by varying the substituents on sulfur and salicylaldehyde, and by changing the metal ion, a tuning of the properties of the corresponding complexes can be achieved in principle. As shown in Fig. 1 (*b*), *S*-alkylisothiosemicarbazones can react with aldehydes. The template reaction of *S*-alkylisothiosemicarbazones with substituted salicyladehydes, in the presence of metal ions fitted for square planar coordination (*e. g.* VO(II), Cu(II), Ni(II), UO_2_(II)) can lead to tetradentate (N, N, O, O) complexes, Fig. 1(*b*).

Following our interest in the synthesis and structural characterization of organic and metallorganic compounds containing heterocycles for applications as advanced materials and bioactive compounds (Centore, Ricciotti *et al.*, 2012; Takjoo *et al.*, 2011; Takjoo & Centore, 2013), and in the analysis of crystal structures controlled by the formation of H bonds (Centore *et al.*, 2013), we report the structural investigation of the title compound, (I). (I) was obtained by the template reaction of 5-bromo-2-hydroxybenzaldehyde-*S*- ethylisothiosemicarbazone with salicylaldehyde, in the presence of uranyl acetate.

The molecular structure of (I) is shown in Fig. 2. The heptacoordination around the metal atom can be described as distorted pentagonal bipyramid. The equatorial plane is occupied by the four donor atoms of the tetradentate chelate ligand (N, N, O, O) and by the oxygen donor atom of the coordinated ethanol molecule. The axial positions are occupied by the uranyl oxygen atoms. The bond lengths between uranium and the equatorial donors range between 2.221 (4) Å and 2.576 (4) Å, while the two bond lengths within the uranyl group are significantly shorter (1.764 (4) Å and 1.771 (3) Å). In the equatorial plane, two six-membered and one five-membered rings are formed involving the metal atom. The five membered ring is almost planar, while the two six-membered rings are in envelope conformation, with the metal atom out of the plane. The bite angles corresponding to the formation of the six-membered rings are slightly larger than the five membered ring.

Molecules of the title compound have H bonding donor and acceptor groups, and the crystal packing shows the formation of H bonds. In the crystal, molecules form centrosymmetric dimers through H bonding between O–H donors and phenolato O^-^ acceptors, giving rise to ring patterns *R*^2^_2_(8). The rings include the uranium atoms, Fig. 3. The same pattern is present in the crystals of a closely related compound (Takjoo *et al.*, 2012). The oxygen atoms of the uranyl moiety are involved in weak H bonding interactions. In the case of O3, aromatic and imino C–H are the weak donors, and *R*^1^_2_(6) ring patterns are observed. In the case of O4, methyl and aromatic C–H are the weak donors, Fig. 4.

### Experimental

Preparation of 5-Bromo-2-hydroxybenzaldehyde-*S*-ethylisothiosemicarbazone hydroiodide (H_2_L.HI). A solution of thiosemicarbazide (0.91 g, 10 mmol) in ethanol (5 mL) was treated with ethyliodide (1.55 g, 10 mmol) and was refluxed for 3 h at 90 °C. 5-Bromo-2-hydroxybenzaldehyde (2.011 g, 10 mmol) was then added to the resulting solution and the reflux was continued for an additional 1 h. A yellow precipitate formed that was filtered off, washed with cold ethanol and dried in vacuum over silica gel. Yield 75%. Mp. 190 °C.

Preparation of the title compound. H_2_L.HI (0.430 g, 1.0 mmol) and salicylaldehyde (0.12 g, 1.0 mmol) were dissolved in warm ethanol (10 mL). To this solution, a solution of UO_2_(OAc)_2_.2H_2_O (0.42 g, 1.0 mmol) in 10 mL ethanol was added. The resulting solution was refluxed for 30 min. By slow evaporation at room temperature, red crystals of the title compound formed after several days. The crystals were collected by filtration, washed with diethyl ether and dried in air. Yield 37%. Mp. 156 °C (dec). Anal. Calc. for C_19_H_20_N_3_O_5_SBrU: C, 31.68; H, 2.80; N, 5.83%. Found: C, 31.43; H, 2.72; N, 5.74%. IR (cm^-1^): ν(C–H) 2931–3004 w; ν(C=N) + ν(C=C) 1604 *vs*, 1527 s; ν(C–O) 1288 s; ν(N–N) 1010 w; ν_asy_(*trans*-UO_2_) 910 s; ν_sy_(*trans*-UO_2_) 877 m. UV-VIS [MeOH, λ_max_/nm (log ε_max_/*M*^-1^ cm^-1^)]: 222 (4.39), 248 (4.34), 312 (4.20), 410 (3.79). Molar conductivity (1.0×10^-3^*M*; MeOH): 6 Ω^-1^ cm^2^ mol^-1^.

### Refinement

The H atom of the hydroxy group was located in difference map. All other H atoms were generated stereochemically and were refined by the riding model. For all H atoms *U*_iso_=1.2×*U*_eq_ of the carrier atom was assumed (1.5 for methyl groups). The crystal structure shows a remarkable degree of static disorder. In fact, molecules enter the crystal in two orientations of the tetradentate ligand, which are nearly obtained by rotation of 180° around the line joining U1 with the barycentre of N2A–C8A. There results a complete superposition of the atoms of the tetradentate ligand in the two orientations, with exception for bromine, sulfur and the methyl group of the *S*-ethyl tail. These atoms were found in difmaps and were refined. By refining also the occupancy factor, it resulted that the main orientation has an occupation factor higher by far than the other (0.836 (2) and 0.164 (2)). The resolved atoms of the low populated split positions were refined with some restraints on bond lengths and angles in order to keep the same geometry of the higher occupancy position (SAME instruction of *SHELXL97*). Also the ethanol molecule coordinated to uranyl is disordered over three positions. Since the methylene carbon atoms of the three different positions of the ethanol molecule are quite close to each other, they were refined with isotropic displacement parameters.

### Crystal data

**Table:**

[U(C_17_H_14_BrN_3_O_2_S)O_2_(C_2_H_6_O)]	*Z* = 2
*M**_r_* = 720.38	*F*(000) = 676
Triclinic, *P*1	*D*_x_ = 2.109 Mg m^−^^3^
*a* = 10.3720 (17) Å	Mo *K*α radiation, λ = 0.71073 Å
*b* = 11.1380 (14) Å	Cell parameters from 110 reflections
*c* = 11.167 (1) Å	θ = 4.6–23.6°
α = 69.428 (10)°	µ = 9.04 mm^−^^1^
β = 86.870 (11)°	*T* = 293 K
γ = 70.379 (10)°	Prism, red
*V* = 1134.7 (3) Å^3^	0.40 × 0.20 × 0.20 mm

### Data collection

**Table:**

Bruker–Nonius KappaCCD diffractometer	5207 independent reflections
Radiation source: normal-focus sealed tube	4347 reflections with *I* > 2σ(*I*)
Graphite monochromator	*R*_int_ = 0.045
Detector resolution: 9 pixels mm^-1^	θ_max_ = 27.5°, θ_min_ = 3.3°
CCD rotation images, thick slices scans	*h* = −13→13
Absorption correction: multi-scan (*SADABS*; Bruker, 2001)	*k* = −14→14
*T*_min_ = 0.123, *T*_max_ = 0.265	*l* = −14→13
15923 measured reflections	

### Refinement

**Table:**

Refinement on *F*^2^	Primary atom site location: structure-invariant direct methods
Least-squares matrix: full	Secondary atom site location: difference Fourier map
*R*[*F*^2^ > 2σ(*F*^2^)] = 0.029	Hydrogen site location: inferred from neighbouring sites
*wR*(*F*^2^) = 0.068	H-atom parameters constrained
*S* = 1.08	*w* = 1/[σ^2^(*F*_o_^2^) + (0.0179*P*)^2^ + 1.5042*P*] where *P* = (*F*_o_^2^ + 2*F*_c_^2^)/3
5207 reflections	(Δ/σ)_max_ = 0.001
306 parameters	Δρ_max_ = 1.02 e Å^−^^3^
53 restraints	Δρ_min_ = −1.22 e Å^−^^3^

### Special details

**Table:**

Geometry. All e.s.d.'s (except the e.s.d. in the dihedral angle between two l.s. planes) are estimated using the full covariance matrix. The cell e.s.d.'s are taken into account individually in the estimation of e.s.d.'s in distances, angles and torsion angles; correlations between e.s.d.'s in cell parameters are only used when they are defined by crystal symmetry. An approximate (isotropic) treatment of cell e.s.d.'s is used for estimating e.s.d.'s involving l.s. planes.
Refinement. Refinement of *F*^2^ against ALL reflections. The weighted *R*-factor *wR* and goodness of fit *S* are based on *F*^2^, conventional *R*-factors *R* are based on *F*, with *F* set to zero for negative *F*^2^. The threshold expression of *F*^2^ > σ(*F*^2^) is used only for calculating *R*-factors(gt) *etc*. and is not relevant to the choice of reflections for refinement. *R*-factors based on *F*^2^ are statistically about twice as large as those based on *F*, and *R*- factors based on ALL data will be even larger.

### Fractional atomic coordinates and isotropic or equivalent isotropic displacement parameters (Å^2^)

**Table:**

	*x*	*y*	*z*	*U*_iso_*/*U*_eq_	Occ. (<1)
C1	0.6222 (5)	−0.4166 (5)	1.0165 (5)	0.0480 (13)	
H1B	0.6535	−0.4967	1.0874	0.058*	0.1644 (18)
Br1A	0.68347 (8)	−0.57406 (7)	1.16068 (8)	0.0615 (2)	0.8356 (18)
Br1B	−0.0277 (5)	0.9058 (5)	0.2778 (5)	0.0882 (18)	0.1644 (18)
C2	0.6697 (6)	−0.4110 (6)	0.8960 (6)	0.0547 (14)	
H2	0.7339	−0.4884	0.8871	0.066*	
C3	0.6239 (5)	−0.2946 (6)	0.7911 (6)	0.0494 (13)	
H3	0.6578	−0.2935	0.7120	0.059*	
C4	0.5257 (5)	−0.1758 (5)	0.8013 (5)	0.0399 (11)	
O1	0.4757 (4)	−0.0655 (3)	0.6971 (3)	0.0439 (8)	
C5	0.4789 (5)	−0.1789 (5)	0.9220 (5)	0.0389 (11)	
C6	0.5280 (5)	−0.3005 (5)	1.0281 (5)	0.0436 (12)	
H6	0.4962	−0.3027	1.1081	0.052*	
C7	0.3759 (5)	−0.0669 (5)	0.9449 (5)	0.0405 (11)	
H7	0.3427	−0.0849	1.0261	0.049*	
N1	0.3248 (4)	0.0560 (4)	0.8644 (4)	0.0395 (9)	
N2A	0.2242 (5)	0.1373 (5)	0.9202 (4)	0.0480 (11)	0.8356 (18)
N2B	0.1747 (5)	0.2653 (5)	0.8539 (5)	0.0441 (11)	0.1644 (18)
C8A	0.1747 (5)	0.2653 (5)	0.8539 (5)	0.0441 (11)	0.8356 (18)
S1A	0.05176 (18)	0.36638 (19)	0.92399 (19)	0.0574 (5)	0.8356 (18)
C16A	0.0450 (13)	0.2473 (13)	1.0815 (13)	0.088 (4)	0.8356 (18)
H16A	0.1376	0.1892	1.1170	0.106*	0.8356 (18)
H16B	0.0039	0.2972	1.1377	0.106*	0.8356 (18)
C17A	−0.0377 (13)	0.1597 (13)	1.0763 (14)	0.128 (5)	0.8356 (18)
H17A	−0.0391	0.0973	1.1611	0.192*	0.8356 (18)
H17B	0.0034	0.1094	1.0215	0.192*	0.8356 (18)
H17C	−0.1299	0.2169	1.0431	0.192*	0.8356 (18)
C8B	0.2242 (5)	0.1373 (5)	0.9202 (4)	0.0480 (11)	0.085 (6)
S1B	0.172 (3)	0.0871 (19)	1.0706 (17)	0.062 (4)*	0.085 (6)
C16B	0.027 (5)	0.231 (5)	1.075 (5)	0.088 (4)	0.085 (6)
H16C	0.0349	0.3161	1.0164	0.106*	0.085 (6)
H16D	−0.0584	0.2236	1.0522	0.106*	0.085 (6)
C17B	0.033 (7)	0.223 (6)	1.213 (5)	0.128 (5)	0.085 (6)
H17D	−0.0425	0.2959	1.2242	0.192*	0.085 (6)
H17E	0.1179	0.2315	1.2326	0.192*	0.085 (6)
H17F	0.0279	0.1372	1.2687	0.192*	0.085 (6)
C8C	0.2242 (5)	0.1373 (5)	0.9202 (4)	0.0480 (11)	0.079 (6)
S1C	0.127 (3)	0.0792 (19)	1.043 (2)	0.062 (4)*	0.079 (6)
C16C	0.027 (5)	0.231 (5)	1.075 (5)	0.088 (4)	0.079 (6)
H16E	0.0571	0.3054	1.0221	0.106*	0.079 (6)
H16F	−0.0683	0.2541	1.0474	0.106*	0.079 (6)
C17C	0.033 (7)	0.223 (6)	1.213 (5)	0.128 (5)	0.079 (6)
H17G	−0.0321	0.3043	1.2208	0.192*	0.079 (6)
H17H	0.1237	0.2152	1.2372	0.192*	0.079 (6)
H17I	0.0116	0.1451	1.2674	0.192*	0.079 (6)
N3	0.2190 (4)	0.3221 (4)	0.7343 (4)	0.0399 (9)	
C9	0.1450 (5)	0.4453 (5)	0.6638 (6)	0.0464 (12)	
H9	0.0654	0.4855	0.6980	0.056*	
C10	0.1708 (5)	0.5257 (5)	0.5413 (6)	0.0446 (12)	
C11	0.0656 (6)	0.6486 (6)	0.4747 (6)	0.0568 (15)	
H11	−0.0168	0.6753	0.5115	0.068*	
C12	0.0856 (7)	0.7270 (5)	0.3570 (7)	0.0654 (17)	
H12A	0.0141	0.8059	0.3125	0.079*	0.8356 (18)
C13	0.2068 (8)	0.6951 (7)	0.3002 (7)	0.0682 (18)	
H13	0.2172	0.7523	0.2193	0.082*	
C14	0.3117 (7)	0.5792 (7)	0.3632 (6)	0.0593 (15)	
H14	0.3943	0.5580	0.3253	0.071*	
C15	0.2972 (6)	0.4909 (6)	0.4848 (6)	0.0460 (12)	
O2	0.3995 (4)	0.3803 (4)	0.5465 (4)	0.0528 (9)	
O3	0.5501 (4)	0.1468 (4)	0.7532 (4)	0.0489 (9)	
O4	0.2827 (3)	0.1828 (4)	0.5463 (4)	0.0466 (8)	
U1	0.415731 (18)	0.164807 (19)	0.650973 (18)	0.03560 (6)	
O5A	0.5794 (4)	0.1272 (4)	0.4954 (4)	0.0483 (9)	0.470 (16)
H5A	0.5486	0.0995	0.4528	0.058*	0.470 (16)
C18A	0.7137 (10)	0.1409 (15)	0.4828 (12)	0.042 (3)*	0.470 (16)
H18A	0.7110	0.2233	0.4958	0.051*	0.470 (16)
H18B	0.7430	0.1464	0.3976	0.051*	0.470 (16)
C19A	0.8114 (16)	0.0191 (16)	0.581 (2)	0.106 (8)	0.470 (16)
H19A	0.9024	0.0233	0.5713	0.159*	0.470 (16)
H19B	0.8096	−0.0622	0.5706	0.159*	0.470 (16)
H19C	0.7849	0.0177	0.6653	0.159*	0.470 (16)
O5B	0.5794 (4)	0.1272 (4)	0.4954 (4)	0.0483 (9)	0.277 (19)
H5B	0.5487	0.0992	0.4535	0.058*	0.277 (19)
C18B	0.683 (2)	0.192 (3)	0.462 (3)	0.072 (9)*	0.277 (19)
H18C	0.7096	0.2071	0.5353	0.086*	0.277 (19)
H18D	0.6448	0.2796	0.3935	0.086*	0.277 (19)
C19B	0.8029 (17)	0.1076 (18)	0.4192 (18)	0.104 (6)*	0.277 (19)
H19D	0.8752	0.1452	0.4103	0.156*	0.277 (19)
H19E	0.7799	0.1053	0.3382	0.156*	0.277 (19)
H19F	0.8329	0.0167	0.4814	0.156*	0.277 (19)
O5C	0.5794 (4)	0.1272 (4)	0.4954 (4)	0.0483 (9)	0.253 (18)
H5C	0.5476	0.1003	0.4533	0.058*	0.253 (18)
C18C	0.7286 (16)	0.073 (3)	0.531 (2)	0.055 (8)*	0.253 (18)
H18E	0.7482	0.1098	0.5917	0.067*	0.253 (18)
H18F	0.7579	−0.0258	0.5721	0.067*	0.253 (18)
C19C	0.8029 (17)	0.1076 (18)	0.4192 (18)	0.104 (6)*	0.253 (18)
H19G	0.8995	0.0713	0.4436	0.156*	0.253 (18)
H19H	0.7750	0.2050	0.3796	0.156*	0.253 (18)
H19I	0.7840	0.0700	0.3597	0.156*	0.253 (18)

### Atomic displacement parameters (Å^2^)

**Table:**

	*U*^11^	*U*^22^	*U*^33^	*U*^12^	*U*^13^	*U*^23^
C1	0.047 (3)	0.045 (3)	0.043 (3)	−0.007 (2)	−0.012 (2)	−0.011 (2)
Br1A	0.0653 (5)	0.0427 (4)	0.0608 (5)	−0.0068 (3)	−0.0091 (4)	−0.0090 (3)
Br1B	0.083 (3)	0.083 (3)	0.087 (4)	−0.036 (3)	−0.015 (3)	−0.005 (3)
C2	0.046 (3)	0.053 (3)	0.060 (4)	0.000 (3)	−0.001 (3)	−0.029 (3)
C3	0.047 (3)	0.050 (3)	0.047 (3)	−0.001 (2)	0.005 (2)	−0.027 (3)
C4	0.041 (3)	0.043 (3)	0.038 (3)	−0.011 (2)	0.001 (2)	−0.019 (2)
O1	0.056 (2)	0.0418 (19)	0.0314 (18)	−0.0056 (16)	0.0018 (16)	−0.0199 (15)
C5	0.038 (2)	0.044 (3)	0.038 (3)	−0.010 (2)	0.000 (2)	−0.021 (2)
C6	0.048 (3)	0.045 (3)	0.037 (3)	−0.011 (2)	−0.001 (2)	−0.017 (2)
C7	0.044 (3)	0.045 (3)	0.031 (3)	−0.011 (2)	0.005 (2)	−0.016 (2)
N1	0.040 (2)	0.042 (2)	0.035 (2)	−0.0048 (18)	0.0051 (17)	−0.0205 (18)
N2A	0.046 (2)	0.054 (3)	0.044 (3)	−0.008 (2)	0.014 (2)	−0.027 (2)
N2B	0.041 (3)	0.049 (3)	0.046 (3)	−0.010 (2)	0.009 (2)	−0.027 (2)
C8A	0.041 (3)	0.049 (3)	0.046 (3)	−0.010 (2)	0.009 (2)	−0.027 (2)
S1A	0.0515 (10)	0.0548 (10)	0.0647 (12)	−0.0065 (8)	0.0199 (8)	−0.0330 (9)
C16A	0.098 (7)	0.074 (6)	0.097 (7)	−0.026 (5)	0.059 (5)	−0.046 (5)
C17A	0.147 (11)	0.111 (9)	0.149 (13)	−0.061 (9)	0.077 (10)	−0.066 (9)
C8B	0.046 (2)	0.054 (3)	0.044 (3)	−0.008 (2)	0.014 (2)	−0.027 (2)
C16B	0.098 (7)	0.074 (6)	0.097 (7)	−0.026 (5)	0.059 (5)	−0.046 (5)
C17B	0.147 (11)	0.111 (9)	0.149 (13)	−0.061 (9)	0.077 (10)	−0.066 (9)
C8C	0.046 (2)	0.054 (3)	0.044 (3)	−0.008 (2)	0.014 (2)	−0.027 (2)
C16C	0.098 (7)	0.074 (6)	0.097 (7)	−0.026 (5)	0.059 (5)	−0.046 (5)
C17C	0.147 (11)	0.111 (9)	0.149 (13)	−0.061 (9)	0.077 (10)	−0.066 (9)
N3	0.039 (2)	0.042 (2)	0.043 (2)	−0.0112 (18)	0.0030 (18)	−0.0221 (19)
C9	0.037 (3)	0.044 (3)	0.061 (4)	−0.007 (2)	0.002 (2)	−0.028 (3)
C10	0.045 (3)	0.039 (3)	0.054 (3)	−0.014 (2)	−0.001 (2)	−0.019 (2)
C11	0.050 (3)	0.048 (3)	0.069 (4)	−0.012 (3)	−0.009 (3)	−0.020 (3)
C12	0.073 (4)	0.052 (4)	0.064 (4)	−0.023 (3)	−0.015 (3)	−0.006 (3)
C13	0.083 (5)	0.067 (4)	0.054 (4)	−0.036 (4)	−0.005 (3)	−0.008 (3)
C14	0.067 (4)	0.064 (4)	0.055 (4)	−0.034 (3)	0.010 (3)	−0.021 (3)
C15	0.048 (3)	0.045 (3)	0.052 (3)	−0.020 (2)	0.000 (2)	−0.021 (3)
O2	0.046 (2)	0.047 (2)	0.067 (3)	−0.0162 (17)	0.0111 (19)	−0.0222 (19)
O3	0.0410 (19)	0.068 (2)	0.045 (2)	−0.0157 (18)	0.0043 (16)	−0.0310 (19)
O4	0.0400 (18)	0.054 (2)	0.049 (2)	−0.0104 (16)	−0.0014 (16)	−0.0258 (18)
U1	0.03288 (9)	0.04260 (11)	0.03333 (10)	−0.00789 (7)	0.00249 (6)	−0.02045 (7)
O5A	0.0432 (19)	0.071 (3)	0.044 (2)	−0.0205 (18)	0.0099 (16)	−0.0346 (19)
C19A	0.057 (9)	0.090 (13)	0.17 (2)	−0.011 (9)	−0.029 (12)	−0.051 (14)
O5B	0.0432 (19)	0.071 (3)	0.044 (2)	−0.0205 (18)	0.0099 (16)	−0.0346 (19)
O5C	0.0432 (19)	0.071 (3)	0.044 (2)	−0.0205 (18)	0.0099 (16)	−0.0346 (19)

### Geometric parameters (Å, º)

**Table:**

C1—C6	1.378 (7)	C9—H9	0.9300
C1—C2	1.395 (8)	C10—C11	1.410 (7)
C1—Br1A	1.858 (5)	C10—C15	1.417 (8)
C1—H1B	0.9300	C11—C12	1.348 (9)
C2—C3	1.364 (8)	C11—H11	0.9300
C2—H2	0.9300	C12—C13	1.372 (10)
C3—C4	1.410 (7)	C12—H12A	0.9300
C3—H3	0.9300	C13—C14	1.360 (9)
C4—O1	1.328 (6)	C13—H13	0.9300
C4—C5	1.400 (7)	C14—C15	1.404 (8)
O1—U1	2.295 (3)	C14—H14	0.9300
C5—C6	1.405 (7)	C15—O2	1.312 (7)
C5—C7	1.436 (7)	O2—U1	2.221 (4)
C6—H6	0.9300	O3—U1	1.764 (4)
C7—N1	1.290 (6)	O4—U1	1.771 (3)
C7—H7	0.9300	U1—O5A	2.406 (3)
N1—N2A	1.410 (5)	O5A—C18A	1.445 (10)
N1—U1	2.556 (4)	O5A—H5A	0.7809
S1A—C16A	1.808 (13)	O5A—H5B	0.7768
C16A—C17A	1.517 (9)	O5A—H5C	0.7761
C16A—H16A	0.9700	C18A—C19A	1.492 (9)
C16A—H16B	0.9700	C18A—H18A	0.9700
C17A—H17A	0.9600	C18A—H18B	0.9700
C17A—H17B	0.9600	C19A—H19A	0.9600
C17A—H17C	0.9600	C19A—H19B	0.9600
S1B—C16B	1.81 (3)	C19A—H19C	0.9600
C16B—C17B	1.52 (2)	C18B—C19B	1.455 (18)
C16B—H16C	0.9700	C18B—H18C	0.9700
C16B—H16D	0.9700	C18B—H18D	0.9700
C17B—H17D	0.9600	C19B—H19D	0.9600
C17B—H17E	0.9600	C19B—H19E	0.9600
C17B—H17F	0.9600	C19B—H19F	0.9600
N3—C9	1.301 (7)	C18C—H18E	0.9700
N3—U1	2.576 (4)	C18C—H18F	0.9700
C9—C10	1.416 (8)		
			
C6—C1—C2	118.4 (5)	C13—C12—H12A	118.6
C6—C1—Br1A	119.5 (4)	C14—C13—C12	119.4 (6)
C2—C1—Br1A	122.1 (4)	C14—C13—H13	120.3
C6—C1—H1B	120.8	C12—C13—H13	120.3
C2—C1—H1B	120.8	C13—C14—C15	121.0 (6)
Br1A—C1—H1B	1.4	C13—C14—H14	119.5
C3—C2—C1	121.4 (5)	C15—C14—H14	119.5
C3—C2—H2	119.3	O2—C15—C14	120.9 (5)
C1—C2—H2	119.3	O2—C15—C10	120.6 (5)
C2—C3—C4	120.7 (5)	C14—C15—C10	118.5 (5)
C2—C3—H3	119.7	C15—O2—U1	133.4 (3)
C4—C3—H3	119.7	O3—U1—O4	179.05 (17)
O1—C4—C5	121.2 (4)	O3—U1—O2	89.52 (17)
O1—C4—C3	120.1 (5)	O4—U1—O2	90.23 (16)
C5—C4—C3	118.6 (5)	O3—U1—O1	93.70 (16)
C4—O1—U1	134.9 (3)	O4—U1—O1	86.22 (15)
C4—C5—C6	119.3 (4)	O2—U1—O1	159.51 (13)
C4—C5—C7	124.2 (5)	O3—U1—O5A	88.85 (14)
C6—C5—C7	116.3 (5)	O4—U1—O5A	90.21 (14)
C1—C6—C5	121.5 (5)	O2—U1—O5A	82.02 (14)
C1—C6—H6	119.2	O1—U1—O5A	77.82 (13)
C5—C6—H6	119.2	O3—U1—N1	82.19 (16)
N1—C7—C5	126.6 (5)	O4—U1—N1	98.66 (16)
N1—C7—H7	116.7	O2—U1—N1	130.39 (14)
C5—C7—H7	116.7	O1—U1—N1	70.10 (12)
C7—N1—N2A	110.6 (4)	O5A—U1—N1	145.93 (13)
C7—N1—U1	127.6 (3)	O3—U1—N3	97.53 (15)
N2A—N1—U1	120.6 (3)	O4—U1—N3	83.25 (14)
C17A—C16A—S1A	111.4 (10)	O2—U1—N3	70.53 (14)
C17A—C16A—H16A	109.4	O1—U1—N3	128.83 (13)
S1A—C16A—H16A	109.4	O5A—U1—N3	151.69 (14)
C17A—C16A—H16B	109.4	N1—U1—N3	62.37 (13)
S1A—C16A—H16B	109.4	C18A—O5A—U1	129.7 (6)
H16A—C16A—H16B	108.0	C18A—O5A—H5A	123.9
C16A—C17A—H17A	109.5	U1—O5A—H5A	106.3
C16A—C17A—H17B	109.5	C18A—O5A—H5B	124.2
H17A—C17A—H17B	109.5	U1—O5A—H5B	106.0
C16A—C17A—H17C	109.5	H5A—O5A—H5B	0.4
H17A—C17A—H17C	109.5	C18A—O5A—H5C	124.8
H17B—C17A—H17C	109.5	U1—O5A—H5C	105.4
C17B—C16B—S1B	104 (2)	H5A—O5A—H5C	0.9
C17B—C16B—H16C	111.0	H5B—O5A—H5C	1.0
S1B—C16B—H16C	111.0	O5A—C18A—C19A	108.0 (10)
C17B—C16B—H16D	111.0	O5A—C18A—H18A	110.1
S1B—C16B—H16D	111.0	C19A—C18A—H18A	110.1
H16C—C16B—H16D	109.0	O5A—C18A—H18B	110.1
C16B—C17B—H17D	109.5	C19A—C18A—H18B	110.1
C16B—C17B—H17E	109.5	H18A—C18A—H18B	108.4
H17D—C17B—H17E	109.5	C18A—C19A—H19A	109.5
C16B—C17B—H17F	109.5	C18A—C19A—H19B	109.5
H17D—C17B—H17F	109.5	H19A—C19A—H19B	109.5
H17E—C17B—H17F	109.5	C18A—C19A—H19C	109.5
C9—N3—U1	123.7 (4)	H19A—C19A—H19C	109.5
N3—C9—C10	127.9 (5)	H19B—C19A—H19C	109.5
N3—C9—H9	116.0	C19B—C18B—H18C	109.4
C10—C9—H9	116.0	C19B—C18B—H18D	109.4
C11—C10—C9	117.6 (5)	H18C—C18B—H18D	108.0
C11—C10—C15	118.9 (6)	C18B—C19B—H19D	109.5
C9—C10—C15	123.5 (5)	C18B—C19B—H19E	109.5
C12—C11—C10	119.3 (6)	H19D—C19B—H19E	109.5
C12—C11—H11	120.3	C18B—C19B—H19F	109.5
C10—C11—H11	120.3	H19D—C19B—H19F	109.5
C11—C12—C13	122.8 (6)	H19E—C19B—H19F	109.5
C11—C12—H12A	118.6	H18E—C18C—H18F	108.1
			
C6—C1—C2—C3	−0.6 (9)	C15—O2—U1—O4	31.2 (5)
Br1A—C1—C2—C3	−179.9 (5)	C15—O2—U1—O1	111.0 (5)
C1—C2—C3—C4	−0.5 (9)	C15—O2—U1—O5A	121.4 (5)
C2—C3—C4—O1	−175.9 (5)	C15—O2—U1—N1	−70.4 (5)
C2—C3—C4—C5	1.7 (8)	C15—O2—U1—N3	−51.5 (5)
C5—C4—O1—U1	40.8 (7)	C4—O1—U1—O3	36.0 (5)
C3—C4—O1—U1	−141.7 (4)	C4—O1—U1—O4	−144.9 (5)
O1—C4—C5—C6	175.9 (5)	C4—O1—U1—O2	134.6 (5)
C3—C4—C5—C6	−1.7 (8)	C4—O1—U1—O5A	124.0 (5)
O1—C4—C5—C7	−0.2 (8)	C4—O1—U1—N1	−44.3 (4)
C3—C4—C5—C7	−177.8 (5)	C4—O1—U1—N3	−66.7 (5)
C2—C1—C6—C5	0.5 (8)	C7—N1—U1—O3	−68.2 (4)
Br1A—C1—C6—C5	179.9 (4)	N2A—N1—U1—O3	98.1 (4)
C4—C5—C6—C1	0.6 (8)	C7—N1—U1—O4	111.4 (4)
C7—C5—C6—C1	177.0 (5)	N2A—N1—U1—O4	−82.3 (4)
C4—C5—C7—N1	−9.6 (9)	C7—N1—U1—O2	−150.8 (4)
C6—C5—C7—N1	174.2 (5)	N2A—N1—U1—O2	15.5 (4)
C5—C7—N1—N2A	178.9 (5)	C7—N1—U1—O1	28.7 (4)
C5—C7—N1—U1	−13.7 (8)	N2A—N1—U1—O1	−165.0 (4)
U1—N3—C9—C10	−13.0 (8)	C7—N1—U1—O5A	8.0 (6)
N3—C9—C10—C11	170.5 (5)	N2A—N1—U1—O5A	174.3 (3)
N3—C9—C10—C15	−11.3 (9)	C7—N1—U1—N3	−170.9 (5)
C9—C10—C11—C12	−179.0 (6)	N2A—N1—U1—N3	−4.6 (3)
C15—C10—C11—C12	2.7 (8)	C9—N3—U1—O3	117.9 (4)
C10—C11—C12—C13	−2.6 (10)	C9—N3—U1—O4	−61.6 (4)
C11—C12—C13—C14	0.8 (11)	C9—N3—U1—O2	31.0 (4)
C12—C13—C14—C15	0.7 (10)	C9—N3—U1—O1	−141.2 (4)
C13—C14—C15—O2	−178.7 (6)	C9—N3—U1—O5A	16.2 (6)
C13—C14—C15—C10	−0.5 (9)	C9—N3—U1—N1	−165.1 (4)
C11—C10—C15—O2	177.0 (5)	O3—U1—O5A—C18A	−20.1 (8)
C9—C10—C15—O2	−1.1 (8)	O4—U1—O5A—C18A	159.7 (8)
C11—C10—C15—C14	−1.2 (8)	O2—U1—O5A—C18A	69.5 (8)
C9—C10—C15—C14	−179.4 (5)	O1—U1—O5A—C18A	−114.2 (8)
C14—C15—O2—U1	−133.5 (5)	N1—U1—O5A—C18A	−94.4 (8)
C10—C15—O2—U1	48.3 (7)	N3—U1—O5A—C18A	83.7 (8)
C15—O2—U1—O3	−149.7 (5)	U1—O5A—C18A—C19A	77.3 (14)

### Hydrogen-bond geometry (Å, º)

**Table:**

*D*—H···*A*	*D*—H	H···*A*	*D*···*A*	*D*—H···*A*
O5*A*—H5*A*···O1^i^	0.78	1.89	2.618 (5)	155
C7—H7···O3^ii^	0.93	2.53	3.235 (6)	133
C6—H6···O3^ii^	0.93	2.63	3.368 (6)	137
C11—H11···O4^iii^	0.93	2.66	3.443 (7)	143
C19*A*—H19*B*···O4^i^	0.96	2.58	3.451 (19)	151

Symmetry codes: (i) −*x*+1, −*y*, −*z*+1; (ii) −*x*+1, −*y*, −*z*+2; (iii) −*x*, −*y*+1, −*z*+1.
